# Supergiant Facial Basal Cell Carcinoma With Orbital Involvement: A Preventable Consequence of Delayed Diagnosis and Limited Access to Care

**DOI:** 10.7759/cureus.108518

**Published:** 2026-05-08

**Authors:** Jesús Iván Martínez-Ortega, Frida Itzel Rosas-Lezama, Arely Gissell Ramirez Cibrian, Alejandra Nicole Macias Quiroga

**Affiliations:** 1 Histology, Autonomous University of Nuevo Leon, San Nicolás de los Garza, MEX; 2 Dermatology, Dermatology Institute of Jalisco, Zapopan, MEX; 3 Internal Medicine, Instituto Mexicano del Seguro Social (IMSS), Merida, MEX; 4 General Medicine, Universidad Autonoma de Campeche, Campeche, MEX; 5 Dermatology, Hospital Obrero No. 1, La Paz, BOL

**Keywords:** basal cell carcinoma, delayed diagnosis, elderly, giant basal cell carcinoma, healthcare access, health disparities, hedgehog pathway inhibitors, locally advanced cancer, orbital involvement, supergiant basal cell carcinoma

## Abstract

Supergiant basal cell carcinoma is an exceptionally rare presentation of an otherwise highly curable malignancy and is most often associated with prolonged neglect and limited access to care. We report a case of a 94-year-old man with a long-standing infiltrative facial lesion extending from the right preauricular and temporal region to the nasal sidewall and lower eyelid, associated with ectropion. Dermoscopy revealed blue-gray globules and pigmented ovoid nests, along with white structureless areas and a milky-red background. Biopsy confirmed pigmented basal cell carcinoma.

Given advanced local invasion, frailty, and the unavailability of hedgehog pathway inhibitors in the public healthcare system, curative treatment was not feasible, and palliative radiotherapy was initiated. The patient deteriorated and died shortly thereafter; no evidence of metastatic disease was identified, and death was not attributed to basal cell carcinoma. This case highlights how delays in diagnosis and limited access to modern systemic therapies can transform a typically low-mortality cancer into a source of significant morbidity. Strengthening early detection strategies and improving access to guideline-based treatments remain critical to preventing such outcomes.

## Introduction

Basal cell carcinoma (BCC) is the most prevalent form of cancer worldwide, with a steadily increasing incidence. It is characterized by aberrant activation of the Sonic Hedgehog (Hh) signaling pathway, a central driver of tumor development and a key therapeutic target in advanced disease [1,2]. BCC exhibits a broad spectrum of clinical presentations, including uncommon anatomical locations and morphologic variants, as well as rare extreme forms related to prolonged disease evolution [1,3].

Giant BCC, commonly defined as lesions measuring ≥5 cm in diameter, accounts for approximately 0.5-2% of all cases. “Supergiant” basal cell carcinoma (SGBCC), defined as tumors measuring ≥20 cm, represents an exceptionally rare and extreme presentation. These cases are typically associated with prolonged disease evolution and delayed access to medical care rather than intrinsic tumor aggressiveness [1,2]. A recent systematic review identified approximately 20 reported cases worldwide, most of which occurred in men, were located on the trunk, and were present for more than a decade. Facial and orbital involvement are particularly uncommon [1].

## Case presentation

A 94-year-old man with residual neurological deficits following an ischemic stroke was admitted for inpatient care, during which a long-standing facial tumor was identified. According to his daughter, the lesion had been present for at least 20 years, with gradual progression over time. On admission, the patient was hemodynamically stable but frail, with significant functional dependence related to prior neurologic deficits. Basic laboratory studies showed no clinically significant abnormalities aside from mild anemia consistent with chronic disease.

**Figure 1:**
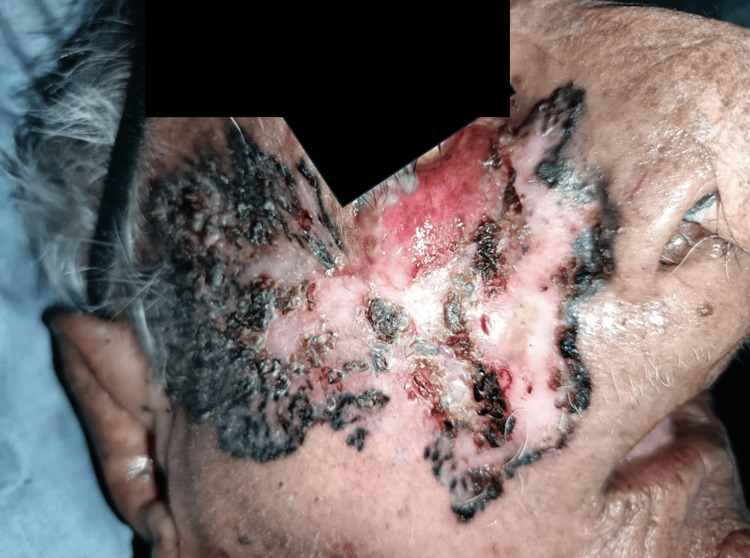
Clinical presentation of supergiant basal cell carcinoma. Extensive ulcerated and exophytic tumor involving the periocular and malar regions, showing marked heterogeneity with areas of ulceration, hemorrhagic crusting, erythema, and irregular black-to-brown pigmentation. The lesion demonstrates a mixture of necrotic, vascular, and pigmented components, consistent with advanced basal cell carcinoma.

Physical examination revealed a massive, infiltrative plaque-like lesion extending from the right preauricular and temporal region to the nasal sidewall and lower eyelid, measuring approximately 20×8 cm. The lesion exhibited extensive ulceration, hemorrhagic crusting, erythema, and irregular black-to-brown pigmentation, with associated lower eyelid ectropion. No palpable regional lymphadenopathy was identified (Figure 1).

**Figure 2:**
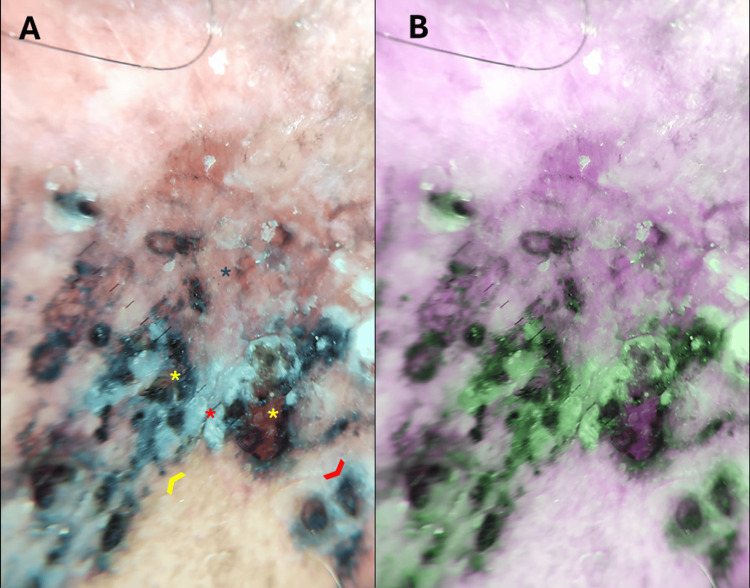
Dermoscopic features and color-enhanced visualization. (A) Dermoscopic image showing multiple pigment-associated structures, including blue-gray globules (yellow arrowheads) and pigmented ovoid nests (red arrowheads). White structureless areas suggestive of keratin are indicated (red asterisk), while focal ulceration and crusting are marked (yellow asterisks). Focal milky-red areas corresponding to vascular components are present (blue asterisk). Arborizing vessels are not clearly identifiable, likely due to ulceration and architectural disorganization. (B) Color-enhanced image generated to improve visualization of chromatic heterogeneity. Regions corresponding to vascular components are relatively accentuated in purple-magenta hues, whereas pigment-associated areas appear darker or greenish, facilitating visual differentiation between vascular and non-vascular chromatic components. Image processing and visualization were performed using Fiji (ImageJ; Bethesda, MD: National Institutes of Health).

**Figure 3:**
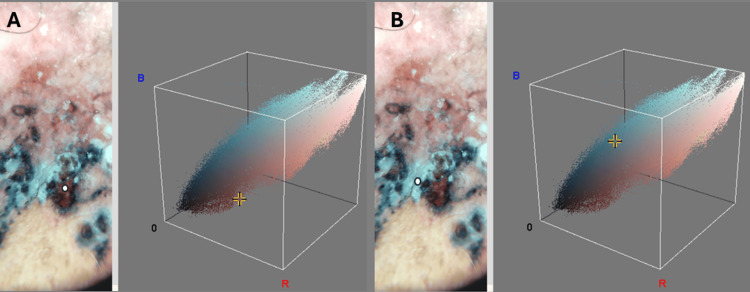
Chromatic distribution of selected dermoscopic regions. Representative sampling of dermoscopic areas demonstrating differences in chromatic composition. Points corresponding to vascular (erythematous) and pigment-associated (blue-gray) regions occupy distinct positions within the color space, illustrating the heterogeneity of color distribution. Axes represent color channels (R: red; B: blue). This visualization supports the presence of distinct underlying structural components. Image processing and visualization were performed using Fiji (ImageJ; Bethesda, MD: National Institutes of Health).

Dermoscopy revealed multiple blue-gray globules and pigmented ovoid nests, consistent with pigment-associated structures, along with white structureless areas suggestive of keratin or fibrosis, and a milky-red background corresponding to vascular components. Areas of ulceration and crusting were evident. Arborizing vessels were not clearly identifiable, likely due to tumor ulceration and architectural disorganization. Overall, the lesion demonstrated marked chromatic heterogeneity with coexisting vascular and pigment-associated features (Figures 2A, 2B, 3A, 3B). To further illustrate this heterogeneity, color-enhanced visualization allowed clearer differentiation between vascular and pigment-associated regions, supporting the presence of distinct structural components within the lesion (Figures 2B, 3A, 3B).

**Figure 4:**
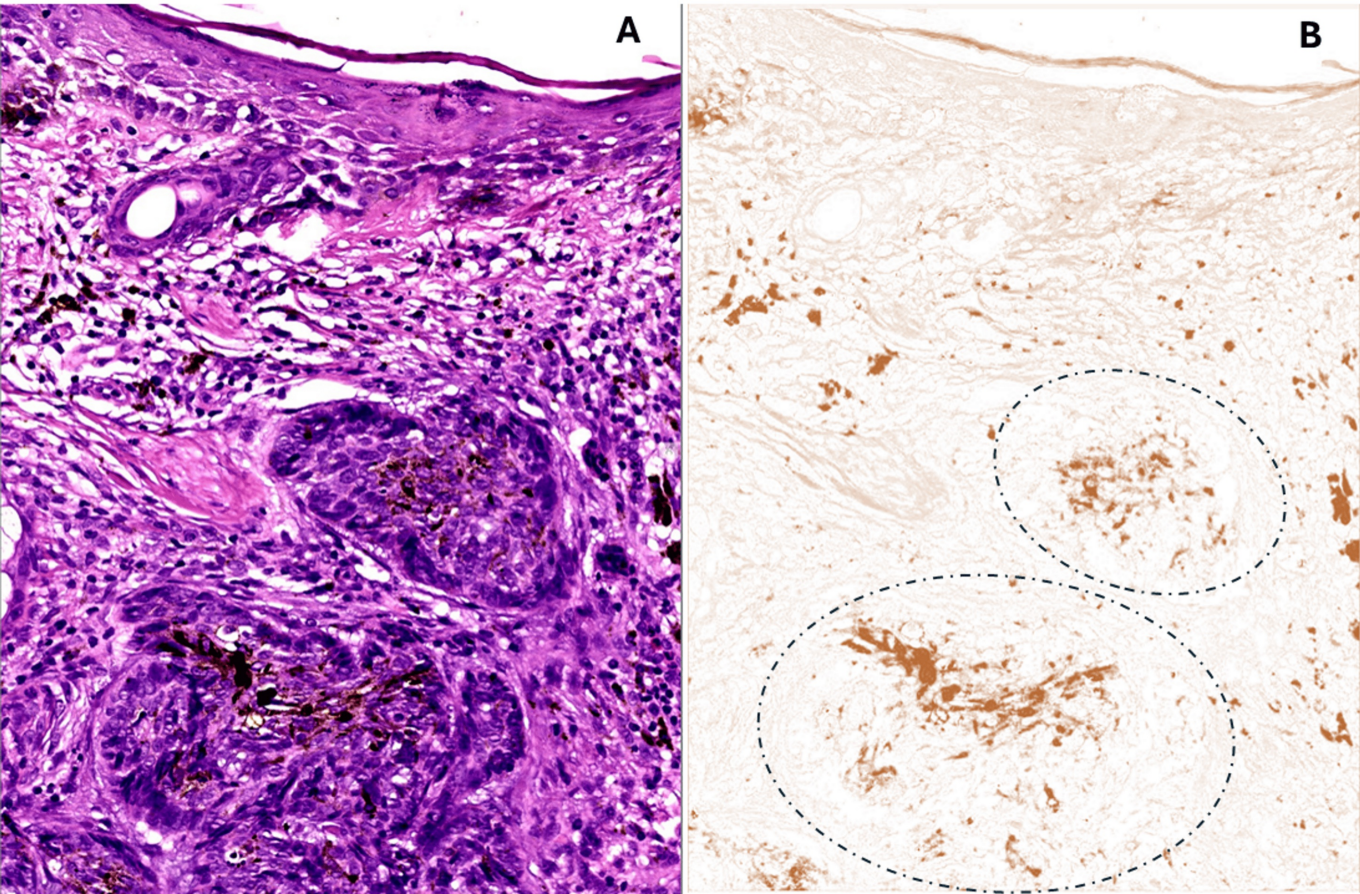
Histopathological features and pigment-enhanced visualization. (A) Hematoxylin-eosin staining showing basaloid tumor nests with peripheral palisading and characteristic peritumoral retraction clefts, embedded within a fibromyxoid stroma. Prominent melanin deposition is observed within tumor nests and in the surrounding stroma, including abundant melanophages. (B) Color-deconvolution image, enhancing visualization of pigment distribution. Dotted outlines highlight areas corresponding to tumor nests, approximating tumor architecture and demonstrating increased concentration of pigment within and around these structures. This supports the correlation between dermoscopic blue-gray structures and histopathological pigment localization. Image processing and visualization were performed using Fiji (ImageJ; Bethesda, MD: National Institutes of Health).

Incisional biopsy demonstrated basaloid tumor nests with peripheral palisading and characteristic peritumoral retraction clefts, embedded within a fibromyxoid stroma. Prominent melanin deposition was observed within tumor nests and in the surrounding stroma, including abundant melanophages, consistent with pigmented basal cell carcinoma. Additional image processing enhanced the visualization of pigment-rich areas, supporting clinicopathological correlation with dermoscopic findings (Figures 4A, 4B).

Given the extent of local invasion, advanced age, frailty, and poor performance status, curative surgical management was deemed unfeasible. Additionally, hedgehog pathway inhibitors (HHIs) and other systemic therapies were unavailable through the public healthcare system. Palliative radiotherapy was therefore selected for symptom control. The patient subsequently experienced clinical deterioration and died shortly thereafter due to an acute myocardial infarction. No evidence of metastatic disease was identified on available imaging studies performed during hospitalization.

## Discussion

Supergiant basal cell carcinoma (SGBCC) lies at the end of the spectrum of neglected basal cell carcinoma (BCC) and is most often associated with prolonged disease duration and delayed access to medical care. A recent systematic review identified approximately 20 reported cases worldwide, most occurring on the trunk and often present for ≥10 years, with social vulnerability frequently noted and metastasis reported in a subset [1]. Facial and orbital SGBCCs are particularly uncommon; published cases describe orbital roof involvement, bone invasion, and even globe destruction [4,5]. The present case adds to this rare subset of facial cases and underscores the destructive consequences of delayed diagnosis.

The term supergiant is a descriptive designation for lesions ≥20 cm and does not correspond to a formal staging category. Contemporary management is better framed within advanced BCC classifications, including locally advanced (laBCC) or metastatic BCC (mBCC), as well as the European Academy of Dermatology and Venereology (EADO) distinction between easy- and difficult-to-treat tumors, often corresponding to stage IIIB-IIIC in advanced cases. These frameworks guide systemic therapy, with hedgehog pathway inhibitors (HHIs), such as vismodegib or sonidegib, recommended as first-line treatment, and anti-PD-1 therapy (cemiplimab) as second-line in selected cases [6-8].

Importantly, several studies suggest that the development of giant BCC is more closely related to delayed presentation and healthcare access limitations than to intrinsic tumor aggressiveness. A 20-year UK study of 43 patients with giant BCC found that tumor size was significantly correlated with delay in presentation (p=0.03), and that most cases (84%) arose de novo rather than from recurrent disease. Notably, the majority of tumors were not biologically aggressive and responded well to conservative surgical excision, with no recurrences observed among cases treated with ≤1 cm margins during follow-up [9]. Similarly, an Italian multicenter study identified the following two main patient profiles: those with delayed access to medical care and those previously managed with inappropriate treatments, with only a minority attributable to inherently aggressive tumor behavior [10].

However, once BCC progresses to deeply invasive disease, outcomes worsen substantially. A meta-analysis of 311 giant BCC cases reported a disease-specific mortality of 18.4%, with independent predictors of death including tumor diameter (HR: 1.12 per cm), bone invasion (HR: 4.19), brain invasion (HR: 8.23), and distant metastases (HR: 14.48) [11]. A Dutch cohort study of locally advanced BCC reported a five-year disease-specific survival of 79%, compared to only 30% in metastatic disease [12]. Furthermore, tumors ≥4 cm with invasion beyond subcutaneous fat demonstrated markedly increased risks of metastasis and mortality [13].

Beyond tumor biology, patient-related factors such as advanced age, frailty, and comorbidities significantly influence therapeutic decisions. Nevertheless, this case highlights a critical limitation in many resource-constrained settings as follows: the lack of access to HHIs and immunotherapy. In such scenarios, treatment options may be restricted to surgery or palliative radiotherapy; however, in the present case, the patient’s clinical condition and rapid deterioration limited the feasibility of such interventions. This gap between evidence-based recommendations and real-world accessibility remains a major determinant of outcomes. Given that BCC is typically a slow-growing and highly curable malignancy, extreme presentations such as SGBCC should be regarded as preventable failures of early detection, timely referral, and equitable access to modern therapies. Strengthening these elements represents a key opportunity to reduce avoidable morbidity and mortality [6,8,9,12].

Emerging molecular biomarkers may provide additional insight into therapeutic response in advanced basal cell carcinoma. In this context, CD155 expression has been associated with Sonic Hedgehog pathway activation, raising the possibility that circulating soluble CD155 (sCD155) levels could reflect pathway activity and tumor burden. Given that advanced and supergiant BCCs are often candidates for hedgehog pathway inhibitors, such a biomarker could help identify patients more likely to respond to therapy, as well as assist in treatment monitoring and detection of residual or recurrent disease. However, this hypothesis requires validation in larger studies [14,15].

In the present case, the extreme tumor size and prolonged clinical course highlight the impact of delayed recognition and limited access to timely evaluation. While basal cell carcinoma is typically indolent, progression to advanced forms such as SGBCC appears to be largely driven by prolonged disease evolution rather than intrinsic biological aggressiveness.

In this context, approaches that facilitate earlier recognition may be particularly relevant in resource-limited or rural settings. Teledermatology and simplified image-based evaluation strategies have been proposed as tools to support initial assessment and referral. Additionally, emerging image-based analytical methods, including quantitative assessment of color heterogeneity, may provide complementary information to clinical and dermoscopic evaluation. Prior work has demonstrated the feasibility of such approaches using accessible tools such as smartphone-acquired images and ImageJ-based analysis, suggesting a potential role in the evaluation of cutaneous neoplasms [16,17]. While these image-enhanced visualizations are exploratory, they may aid in highlighting subtle chromatic differences and improving recognition of vascular and pigment-associated components, potentially supporting clinical interpretation in selected cases.

## Conclusions

SGBCC should be understood as a preventable extreme of a common and typically indolent malignancy rather than a distinct biological entity. This case highlights how delayed diagnosis and limited access to effective therapies can lead to severe local destruction and adverse outcomes. Improving early detection, streamlining referral pathways, and ensuring equitable access to guideline-based targeted treatments remain key to reducing avoidable morbidity in advanced BCC.
